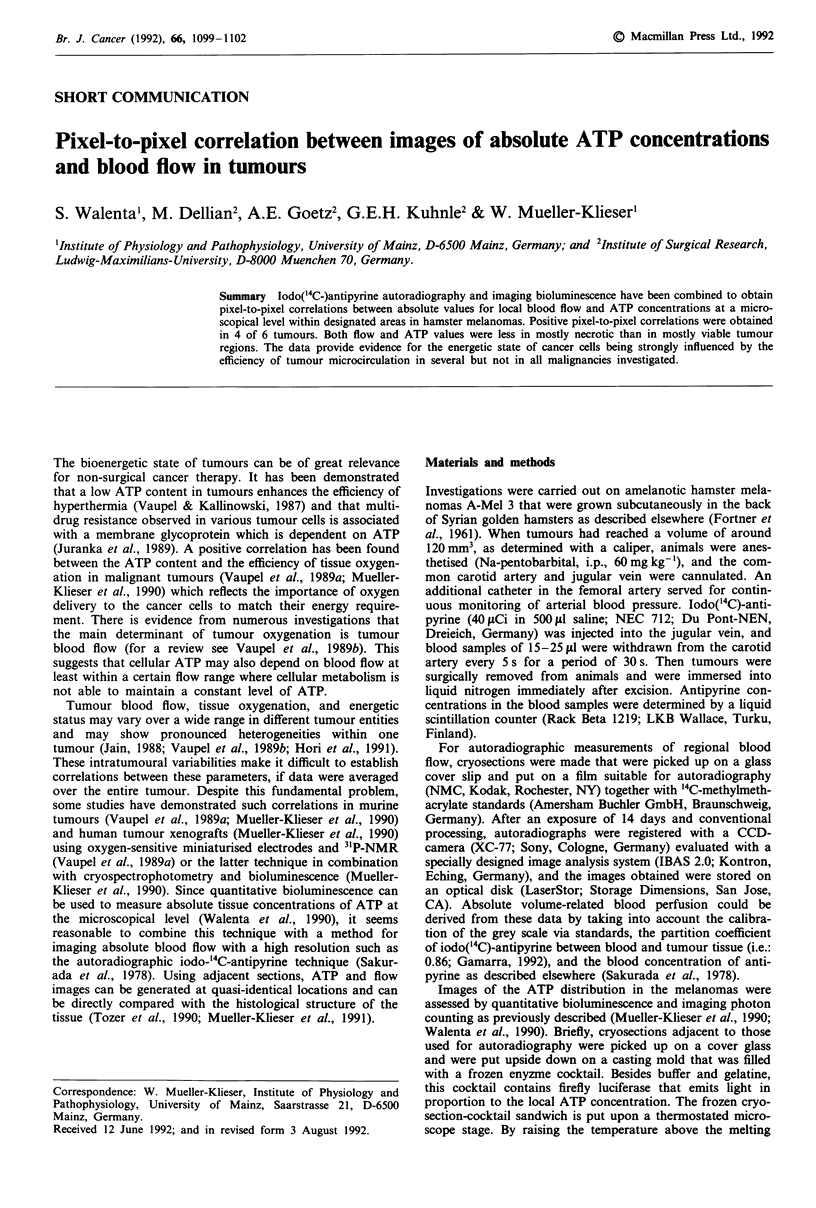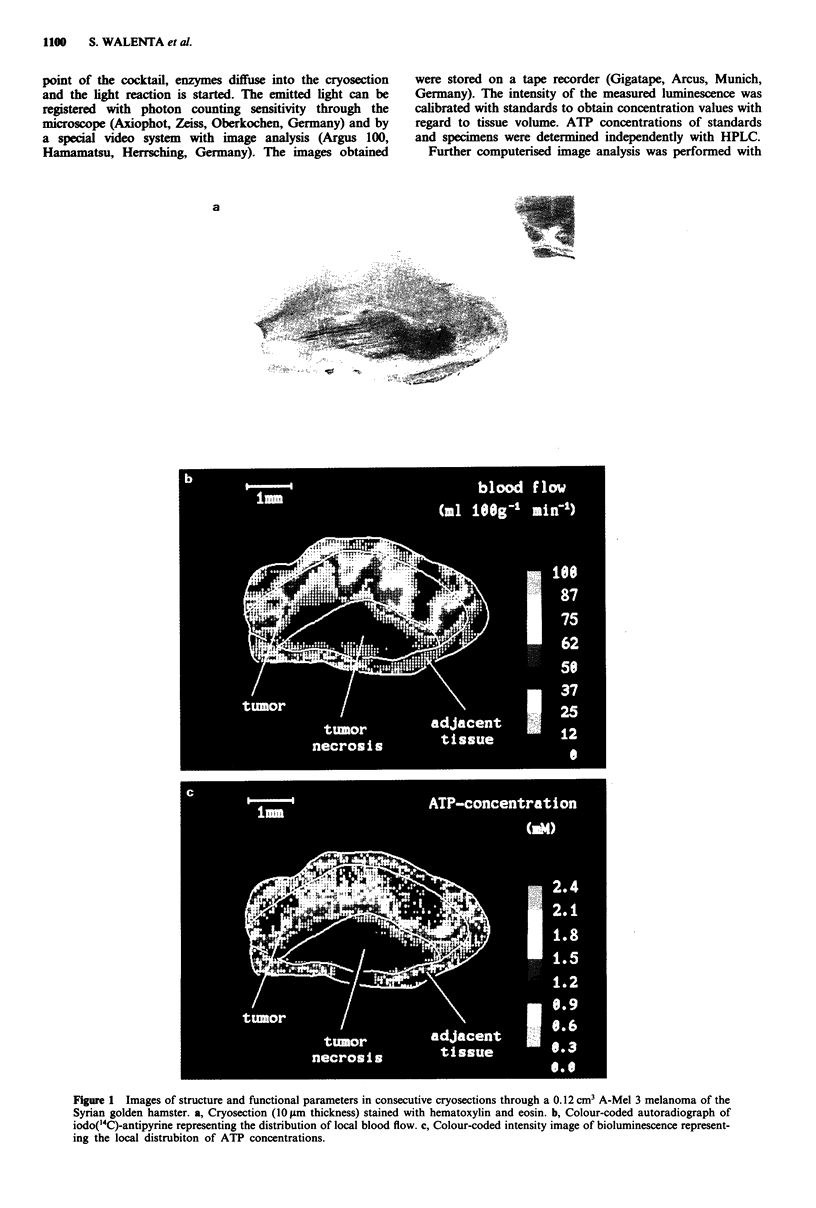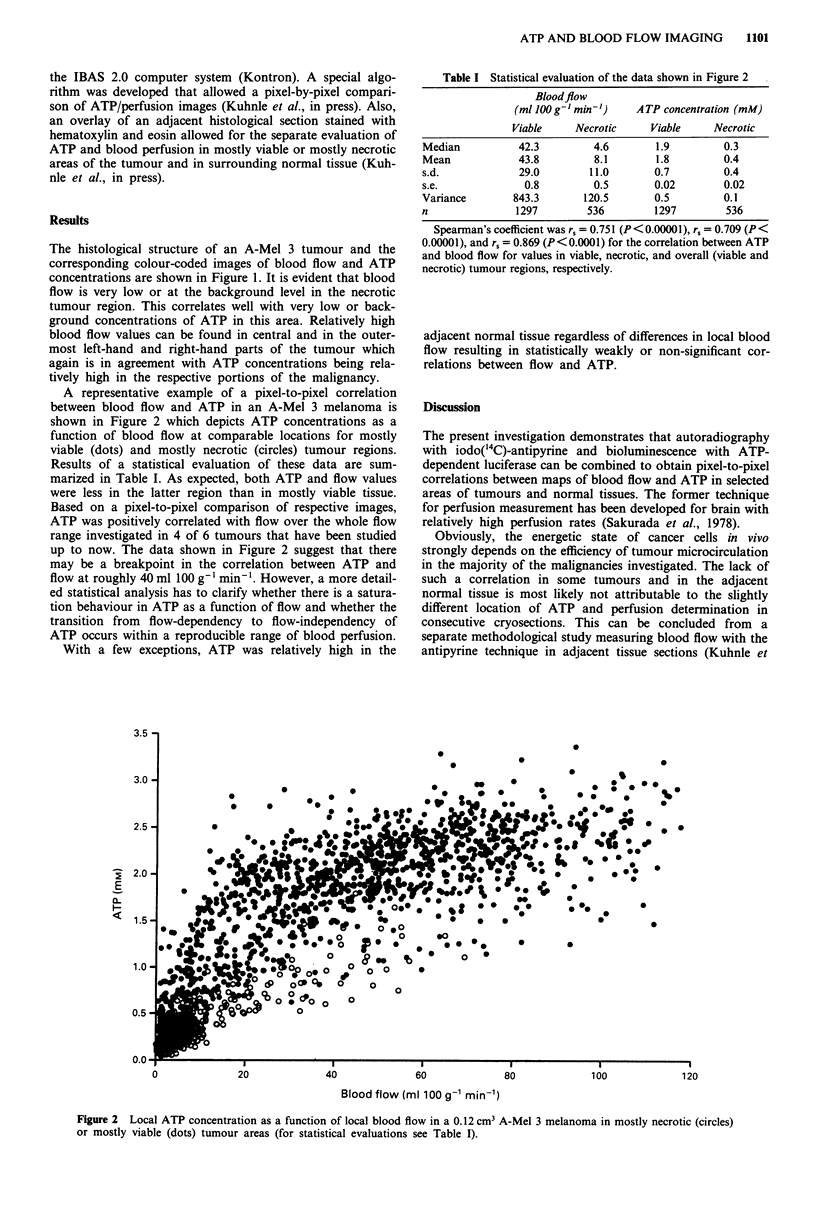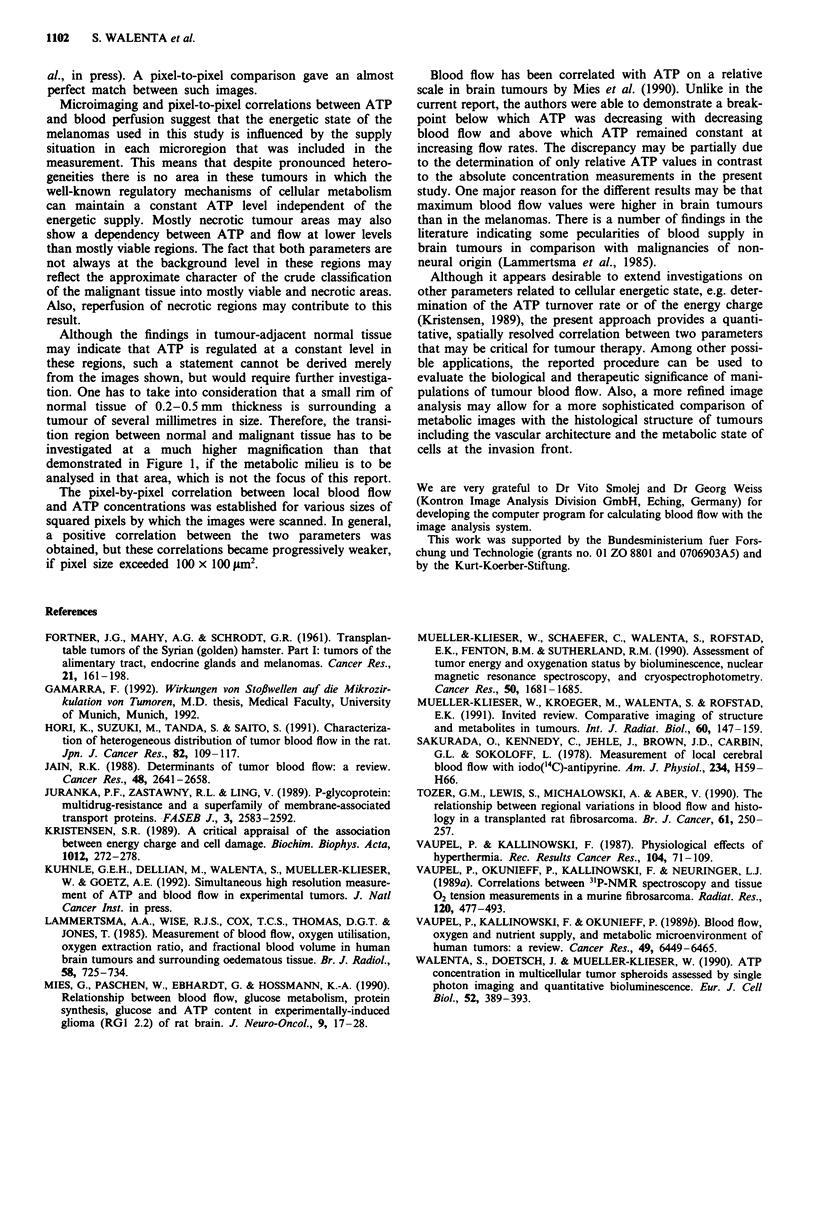# Pixel-to-pixel correlation between images of absolute ATP concentrations and blood flow in tumours.

**DOI:** 10.1038/bjc.1992.417

**Published:** 1992-12

**Authors:** S. Walenta, M. Dellian, A. E. Goetz, G. E. Kuhnle, W. Mueller-Klieser

**Affiliations:** Institute of Physiology and Pathophysiology, University of Mainz, Germany.

## Abstract

**Images:**


Br. J. Cancer (1992), 66, 1099–1102 © Macmillan Press Ltd., 1992

SHORT COMMUNICATION

### Pixel-to-pixel correlation between images of absolute ATP concentrations and blood flow in tumours

S. Walenta[1], M. Dellian[2], A.E. Goetz[2], G.E.H. Kuhnle[2] & W. Mueller-Klieser[1]

[1]*Institute of Physiology and Pathophysiology, University of Mainz, D-6500 Mainz, Germany; and* [2]*Institute of Surgical Research, Ludwig-Maximilians-University, D-8000 Muenchen 70, Germany.*

**Summary** Iodo($^{14}$C-)antipyrine autoradiography and imaging bioluminescence have been combined to obtain pixel-to-pixel correlations between absolute values for local blood flow and ATP concentrations at a microscopical level within designated areas in hamster melanomas. Positive pixel-to-pixel correlations were obtained in 4 of 6 tumours. Both flow and ATP values were less in mostly necrotic than in mostly viable tumour regions. The data provide evidence for the energetic state of cancer cells being strongly influenced by the efficiency of tumour microcirculation in several but not in all malignancies investigated.

The bioenergetic state of tumours can be of great relevance for non-surgical cancer therapy. It has been demonstrated that a low ATP content in tumours enhances the efficiency of hyperthermia (Vaupel & Kallinowski, 1987) and that multidrug resistance observed in various tumour cells is associated with a membrane glycoprotein which is dependent on ATP (Juranka *et al.*, 1989). A positive correlation has been found between the ATP content and the efficiency of tissue oxygenation in malignant tumours (Vaupel *et al.*, 1989*a*; Mueller-Klieser *et al.*, 1990) which reflects the importance of oxygen delivery to the cancer cells to match their energy requirement. There is evidence from numerous investigations that the main determinant of tumour oxygenation is tumour blood flow (for a review see Vaupel *et al.*, 1989*b*). This suggests that cellular ATP may also depend on blood flow at least within a certain flow range where cellular metabolism is not able to maintain a constant level of ATP.

Tumour blood flow, tissue oxygenation, and energetic status may vary over a wide range in different tumour entities and may show pronounced heterogeneities within one tumour (Jain, 1988; Vaupel *et al.*, 1989*b*; Hori *et al.*, 1991). These intratumoural variabilities make it difficult to establish correlations between these parameters, if data were averaged over the entire tumour. Despite this fundamental problem, some studies have demonstrated such correlations in murine tumours (Vaupel *et al.*, 1989*a*; Mueller-Klieser *et al.*, 1990) and human tumour xenografts (Mueller-Klieser *et al.*, 1990) using oxygen-sensitive miniaturised electrodes and $^{31}$P-NMR (Vaupel *et al.*, 1989*a*) or the latter technique in combination with cryospectrophotometry and bioluminescence (Mueller-Klieser *et al.*, 1990). Since quantitative bioluminescence can be used to measure absolute tissue concentrations of ATP at the microscopical level (Walenta *et al.*, 1990), it seems reasonable to combine this technique with a method for imaging absolute blood flow with a high resolution such as the autoradiographic iodo-$^{14}$C-antipyrine technique (Sakurada *et al.*, 1978). Using adjacent sections, ATP and flow images can be generated at quasi-identical locations and can be directly compared with the histological structure of the tissue (Tozer *et al.*, 1990; Mueller-Klieser *et al.*, 1991).

Correspondence: W. Mueller-Klieser, Institute of Physiology and Pathophysiology, University of Mainz, Saarstrasse 21, D-6500 Mainz, Germany.
Received 12 June 1992; and in revised form 3 August 1992.

### Materials and methods

Investigations were carried out on amelanotic hamster melanomas A-Mel 3 that were grown subcutaneously in the back of Syrian golden hamsters as described elsewhere (Fortner *et al.*, 1961). When tumours had reached a volume of around 120 mm$^3$, as determined with a caliper, animals were anesthetised (Na-pentobarbital, i.p., 60 mg kg$^{-1}$), and the common carotid artery and jugular vein were cannulated. An additional catheter in the femoral artery served for continuous monitoring of arterial blood pressure. Iodo($^{14}$C)-antipyrine (40 $\mu$Ci in 500 $\mu$l saline; NEC 712; Du Pont-NEN, Dreieich, Germany) was injected into the jugular vein, and blood samples of 15–25 $\mu$l were withdrawn from the carotid artery every 5 s for a period of 30 s. Then tumours were surgically removed from animals and were immersed into liquid nitrogen immediately after excision. Antipyrine concentrations in the blood samples were determined by a liquid scintillation counter (Rack Beta 1219; LKB Wallace, Turku, Finland).

For autoradiographic measurements of regional blood flow, cryosections were made that were picked up on a glass cover slip and put on a film suitable for autoradiography (NMC, Kodak, Rochester, NY) together with $^{14}$C-methylmethacrylate standards (Amersham Buchler GmbH, Braunschweig, Germany). After an exposure of 14 days and conventional processing, autoradiographs were registered with a CCD-camera (XC-77; Sony, Cologne, Germany) evaluated with a specially designed image analysis system (IBAS 2.0; Kontron, Eching, Germany), and the images obtained were stored on an optical disk (LaserStor; Storage Dimensions, San Jose, CA). Absolute volume-related blood perfusion could be derived from these data by taking into account the calibration of the grey scale via standards, the partition coefficient of iodo($^{14}$C)-antipyrine between blood and tumour tissue (i.e.: 0.86; Gamarra, 1992), and the blood concentration of antipyrine as described elsewhere (Sakurada *et al.*, 1978).

Images of the ATP distribution in the melanomas were assessed by quantitative bioluminescence and imaging photon counting as previously described (Mueller-Klieser *et al.*, 1990; Walenta *et al.*, 1990). Briefly, cryosections adjacent to those used for autoradiography were picked up on a cover glass and were put upside down on a casting mold that was filled with a frozen enyzme cocktail. Besides buffer and gelatine, this cocktail contains firefly luciferase that emits light in proportion to the local ATP concentration. The frozen cryosection-cocktail sandwich is put upon a thermostated microscope stage. By raising the temperature above the melting point of the cocktail, enzymes diffuse into the cryosection and the light reaction is started. The emitted light can be registered with photon counting sensitivity through the microscope (Axiophot, Zeiss, Oberkochen, Germany) and by a special video system with image analysis (Argus 100, Hamamatsu, Herrsching, Germany). The images obtained were stored on a tape recorder (Gigatape, Arcus, Munich, Germany). The intensity of the measured luminescence was calibrated with standards to obtain concentration values with regard to tissue volume. ATP concentrations of standards and specimens were determined independently with HPLC.

Further computerised image analysis was performed with

**Figure 1** Images of structure and functional parameters in consecutive cryosections through a 0.12 cm$^3$ A-Mel 3 melanoma of the Syrian golden hamster. **a**, Cryosection (10 $\mu$m thickness) stained with hematoxylin and eosin. **b**, Colour-coded autoradiograph of iodo($^{14}$C)-antipyrine representing the distribution of local blood flow. **c**, Colour-coded intensity image of bioluminescence representing the local distrubiton of ATP concentrations.

the IBAS 2.0 computer system (Kontron). A special algorithm was developed that allowed a pixel-by-pixel comparison of ATP/perfusion images (Kuhnle *et al.*, in press). Also, an overlay of an adjacent histological section stained with hematoxylin and eosin allowed for the separate evaluation of ATP and blood perfusion in mostly viable or mostly necrotic areas of the tumour and in surrounding normal tissue (Kuhnle *et al.*, in press).

### Results

The histological structure of an A-Mel 3 tumour and the corresponding colour-coded images of blood flow and ATP concentrations are shown in Figure 1. It is evident that blood flow is very low or at the background level in the necrotic tumour region. This correlates well with very low or background concentrations of ATP in this area. Relatively high blood flow values can be found in central and in the outermost left-hand and right-hand parts of the tumour which again is in agreement with ATP concentrations being relatively high in the respective portions of the malignancy.

A representative example of a pixel-to-pixel correlation between blood flow and ATP in an A-Mel 3 melanoma is shown in Figure 2 which depicts ATP concentrations as a function of blood flow at comparable locations for mostly viable (dots) and mostly necrotic (circles) tumour regions. Results of a statistical evaluation of these data are summarized in Table I. As expected, both ATP and flow values were less in the latter region than in mostly viable tissue. Based on a pixel-to-pixel comparison of respective images, ATP was positively correlated with flow over the whole flow range investigated in 4 of 6 tumours that have been studied up to now. The data shown in Figure 2 suggest that there may be a breakpoint in the correlation between ATP and flow at roughly 40 ml 100 $g^{-1}$ $min^{-1}$. However, a more detailed statistical analysis has to clarify whether there is a saturation behaviour in ATP as a function of flow and whether the transition from flow-dependency to flow-independency of ATP occurs within a reproducible range of blood perfusion.

With a few exceptions, ATP was relatively high in the adjacent normal tissue regardless of differences in local blood flow resulting in statistically weakly or non-significant correlations between flow and ATP.

**Table I** Statistical evaluation of the data shown in Figure 2

| | *Blood flow (ml 100 $g^{-1}$ $min^{-1}$)* | | *ATP concentration (mM)* | |
|---|---|---|---|---|
| | *Viable* | *Necrotic* | *Viable* | *Necrotic* |
| Median | 42.3 | 4.6 | 1.9 | 0.3 |
| Mean | 43.8 | 8.1 | 1.8 | 0.4 |
| s.d. | 29.0 | 11.0 | 0.7 | 0.4 |
| s.e. | 0.8 | 0.5 | 0.02 | 0.02 |
| Variance | 843.3 | 120.5 | 0.5 | 0.1 |
| *n* | 1297 | 536 | 1297 | 536 |

Spearman's coefficient was $r_s = 0.751$ ($P < 0.00001$), $r_s = 0.709$ ($P < 0.00001$), and $r_s = 0.869$ ($P < 0.0001$) for the correlation between ATP and blood flow for values in viable, necrotic, and overall (viable and necrotic) tumour regions, respectively.

### Discussion

The present investigation demonstrates that autoradiography with iodo($^{14}$C)-antipyrine and bioluminescence with ATP-dependent luciferase can be combined to obtain pixel-to-pixel correlations between maps of blood flow and ATP in selected areas of tumours and normal tissues. The former technique for perfusion measurement has been developed for brain with relatively high perfusion rates (Sakurada *et al.*, 1978).

Obviously, the energetic state of cancer cells *in vivo* strongly depends on the efficiency of tumour microcirculation in the majority of the malignancies investigated. The lack of such a correlation in some tumours and in the adjacent normal tissue is most likely not attributable to the slightly different location of ATP and perfusion determination in consecutive cryosections. This can be concluded from a separate methodological study measuring blood flow with the antipyrine technique in adjacent tissue sections (Kuhnle *et*

**Figure 2** Local ATP concentration as a function of local blood flow in a 0.12 $cm^3$ A-Mel 3 melanoma in mostly necrotic (circles) or mostly viable (dots) tumour areas (for statistical evaluations see Table I).

al., in press). A pixel-to-pixel comparison gave an almost perfect match between such images.

Microimaging and pixel-to-pixel correlations between ATP and blood perfusion suggest that the energetic state of the melanomas used in this study is influenced by the supply situation in each microregion that was included in the measurement. This means that despite pronounced heterogeneities there is no area in these tumours in which the well-known regulatory mechanisms of cellular metabolism can maintain a constant ATP level independent of the energetic supply. Mostly necrotic tumour areas may also show a dependency between ATP and flow at lower levels than mostly viable regions. The fact that both parameters are not always at the background level in these regions may reflect the approximate character of the crude classification of the malignant tissue into mostly viable and necrotic areas. Also, reperfusion of necrotic regions may contribute to this result.

Although the findings in tumour-adjacent normal tissue may indicate that ATP is regulated at a constant level in these regions, such a statement cannot be derived merely from the images shown, but would require further investigation. One has to take into consideration that a small rim of normal tissue of 0.2–0.5 mm thickness is surrounding a tumour of several millimetres in size. Therefore, the transition region between normal and malignant tissue has to be investigated at a much higher magnification than that demonstrated in Figure 1, if the metabolic milieu is to be analysed in that area, which is not the focus of this report.

The pixel-by-pixel correlation between local blood flow and ATP concentrations was established for various sizes of squared pixels by which the images were scanned. In general, a positive correlation between the two parameters was obtained, but these correlations became progressively weaker, if pixel size exceeded $100 \times 100\,\mu m^2$.

Blood flow has been correlated with ATP on a relative scale in brain tumours by Mies et al. (1990). Unlike in the current report, the authors were able to demonstrate a breakpoint below which ATP was decreasing with decreasing blood flow and above which ATP remained constant at increasing flow rates. The discrepancy may be partially due to the determination of only relative ATP values in contrast to the absolute concentration measurements in the present study. One major reason for the different results may be that maximum blood flow values were higher in brain tumours than in the melanomas. There is a number of findings in the literature indicating some pecularities of blood supply in brain tumours in comparison with malignancies of non-neural origin (Lammertsma et al., 1985).

Although it appears desirable to extend investigations on other parameters related to cellular energetic state, e.g. determination of the ATP turnover rate or of the energy charge (Kristensen, 1989), the present approach provides a quantitative, spatially resolved correlation between two parameters that may be critical for tumour therapy. Among other possible applications, the reported procedure can be used to evaluate the biological and therapeutic significance of manipulations of tumour blood flow. Also, a more refined image analysis may allow for a more sophisticated comparison of metabolic images with the histological structure of tumours including the vascular architecture and the metabolic state of cells at the invasion front.

We are very grateful to Dr Vito Smolej and Dr Georg Weiss (Kontron Image Analysis Division GmbH, Eching, Germany) for developing the computer program for calculating blood flow with the image analysis system.

This work was supported by the Bundesministerium fuer Forschung und Technologie (grants no. 01 ZO 8801 and 0706903A5) and by the Kurt-Koerber-Stiftung.

### References

FORTNER, J.G., MAHY, A.G. & SCHRODT, G.R. (1961). Transplantable tumors of the Syrian (golden) hamster. Part I: tumors of the alimentary tract, endocrine glands and melanomas. *Cancer Res.*, **21**, 161–198.

GAMARRA, F. (1992). *Wirkungen von Stoßwellen auf die Mikrozirkulation von Tumoren*, M.D. thesis, Medical Faculty, University of Munich, Munich, 1992.

HORI, K., SUZUKI, M., TANDA, S. & SAITO, S. (1991). Characterization of heterogeneous distribution of tumor blood flow in the rat. *Jpn. J. Cancer Res.*, **82**, 109–117.

JAIN, R.K. (1988). Determinants of tumor blood flow: a review. *Cancer Res.*, **48**, 2641–2658.

JURANKA, P.F., ZASTAWNY, R.L. & LING, V. (1989). P-glycoprotein: multidrug-resistance and a superfamily of membrane-associated transport proteins. *FASEB J.*, **3**, 2583–2592.

KRISTENSEN, S.R. (1989). A critical appraisal of the association between energy charge and cell damage. *Biochim. Biophys. Acta*, **1012**, 272–278.

KUHNLE, G.E.H., DELLIAN, M., WALENTA, S., MUELLER-KLIESER, W. & GOETZ, A.E. (1992). Simultaneous high resolution measurement of ATP and blood flow in experimental tumors. *J. Natl Cancer Inst.* in press.

LAMMERTSMA, A.A., WISE, R.J.S., COX, T.C.S., THOMAS, D.G.T. & JONES, T. (1985). Measurement of blood flow, oxygen utilisation, oxygen extraction ratio, and fractional blood volume in human brain tumours and surrounding oedematous tissue. *Br. J. Radiol.*, **58**, 725–734.

MIES, G., PASCHEN, W., EBHARDT, G. & HOSSMANN, K.-A. (1990). Relationship between blood flow, glucose metabolism, protein synthesis, glucose and ATP content in experimentally-induced glioma (RG1 2.2) of rat brain. *J. Neuro-Oncol.*, **9**, 17–28.

MUELLER-KLIESER, W., SCHAEFER, C., WALENTA, S., ROFSTAD, E.K., FENTON, B.M. & SUTHERLAND, R.M. (1990). Assessment of tumor energy and oxygenation status by bioluminescence, nuclear magnetic resonance spectroscopy, and cryospectrophotometry. *Cancer Res.*, **50**, 1681–1685.

MUELLER-KLIESER, W., KROEGER, M., WALENTA, S. & ROFSTAD, E.K. (1991). Invited review. Comparative imaging of structure and metabolites in tumours. *Int. J. Radiat. Biol.*, **60**, 147–159.

SAKURADA, O., KENNEDY, C., JEHLE, J., BROWN, J.D., CARBIN, G.L. & SOKOLOFF, L. (1978). Measurement of local cerebral blood flow with iodo($^{14}C$)-antipyrine. *Am. J. Physiol.*, **234**, H59–H66.

TOZER, G.M., LEWIS, S., MICHALOWSKI, A. & ABER, V. (1990). The relationship between regional variations in blood flow and histology in a transplanted rat fibrosarcoma. *Br. J. Cancer*, **61**, 250–257.

VAUPEL, P. & KALLINOWSKI, F. (1987). Physiological effects of hyperthermia. *Rec. Results Cancer Res.*, **104**, 71–109.

VAUPEL, P., OKUNIEFF, P., KALLINOWSKI, F. & NEURINGER, L.J. (1989*a*). Correlations between $^{31}P$-NMR spectroscopy and tissue $O_2$ tension measurements in a murine fibrosarcoma. *Radiat. Res.*, **120**, 477–493.

VAUPEL, P., KALLINOWSKI, F. & OKUNIEFF, P. (1989*b*). Blood flow, oxygen and nutrient supply, and metabolic microenvironment of human tumors: a review. *Cancer Res.*, **49**, 6449–6465.

WALENTA, S., DOETSCH, J. & MUELLER-KLIESER, W. (1990). ATP concentration in multicellular tumor spheroids assessed by single photon imaging and quantitative bioluminescence. *Eur. J. Cell Biol.*, **52**, 389–393.